# Community-level education accelerates the cultural evolution of fertility decline

**DOI:** 10.1098/rspb.2013.2732

**Published:** 2014-03-22

**Authors:** Heidi Colleran, Grazyna Jasienska, Ilona Nenko, Andrzej Galbarczyk, Ruth Mace

**Affiliations:** 1 Department of Anthropology, University College London, London WC1H 0BW, UK; 2 Department of Environmental Health, Faculty of Health Sciences, Jagiellonian University Medical College, Grzegorzecka 20, Krakow 31-531, Poland; 3 Department of Animal and Plant Sciences, University of Sheffield, Sheffield S10 2TN, UK

**Keywords:** fertility decline, cultural transmission, education, community effects

## Abstract

Explaining why fertility declines as populations modernize is a profound theoretical challenge. It remains unclear whether the fundamental drivers are economic or cultural in nature. Cultural evolutionary theory suggests that community-level characteristics, for example average education, can alter how low-fertility preferences are transmitted and adopted. These assumptions have not been empirically tested. Here, we show that community-level education accelerates fertility decline in a way that is neither predicted by individual characteristics, nor by the level of economic modernization in a population. In 22 high-fertility communities in Poland, fertility converged on a smaller family size as average education in the community increased—indeed community-level education had a larger impact on fertility decline than did individual education. This convergence was not driven by educational levels being more homogeneous, but by less educated women having fewer children than expected, and more highly educated social networks, when living among more highly educated neighbours. The average level of education in a community may influence the social partners women interact with, both within and beyond their immediate social environments, altering the reproductive norms they are exposed to. Given a critical mass of highly educated women, less educated neighbours may adopt their reproductive behaviour, accelerating the pace of demographic transition. Individual characteristics alone cannot capture these dynamics and studies relying solely on them may systematically underestimate the importance of cultural transmission in driving fertility declines. Our results are inconsistent with a purely individualistic, rational-actor model of fertility decline and suggest that optimization of reproduction is partly driven by cultural dynamics beyond the individual.

## 1. Introduction

Most of the world's population now lives in countries where fertility rates are plummeting, with a global demographic transition to low fertility projected by the end of this century [1]. Yet explaining why and how fertility declines is a profound theoretical challenge [2,3], because it remains unclear whether the fundamental causes are economic or cultural in nature [2–9]. Do individuals optimize their reproductive output based mainly on their own characteristics, or are their decisions driven by the frequency of a particular behaviour in their local community?

Researchers agree that multi-causal, multi-level processes drive the onset and pace of demographic transitions [4–9]. Nonetheless, most research is focused on either micro or macro levels of analysis, with little integration between the individual predictors of fertility, for example education [10], and the wider socio-cultural influences on reproductive decision-making. Studies comparing individual characteristics across a population often favour economic models of fertility decline; these emphasize the optimization of reproductive output based on the costs and benefits of investing in the ‘quality’ over the quantity of children [11–16]. However, studies comparing neighbouring populations have long suggested that people take their reproductive cues from the behaviour of others, and that social transmission and the diffusion of new cultural norms about fertility may be more important [17,18].

Women's education is the key predictor of fertility decline [10]. And yet, at both an individual and an aggregate level, it is unclear whether education is an economic or a cultural variable [10,11]. Demographers have shown that province-, district- or sampling-area-level education is correlated with faster parity-transition rates [19–22], higher rates of contraceptive uptake [19,23–26] and lower fertility desires [19–24], independent of individual education. Neither micro- nor macro-level analyses explicitly incorporate the most important, intermediate-level social structures that define peoples’ social interactions: their local communities and ego-networks. It remains unknown whether aggregated educational effects on individual fertility are due to social transmission of new cultural norms, or to ecological or economic differences between study areas that might incentivize fertility decline. Strong tests of cultural transmission hypotheses require community-level analysis, which most available sources of demographic data do not provide. Social network research can partly bridge this gap, but research thus far has tended to focus on contraceptive diffusion and not on fertility outcomes [27–30], and little is known about how networks themselves may change in the course of demographic transition. Measuring individual access to contraceptives or healthcare, exposure to migrants or mass media, or macro-level fertility rates may capture the general diffusion of information in a population over time [11], but these do not necessarily capture the interpersonal cultural transmission of fertility norms and behaviour in local contexts. Cultural dynamics operate, by definition, at levels beyond the individual and should therefore be examined at the local community level. We know little about how the characteristics of other people in local villages and networks influence women's fertility outcomes, and the causal mechanisms by which communities may affect individuals have not been well tested.

Cultural evolutionary theory provides plausible mechanistic links between individual decision-making and information flow in populations [31,32]. This theory assumes that the frequency with which individuals in a group embrace different beliefs, or behave, is a good proxy for a cultural/social norm, and further, that summary measures of these frequency distributions (such as the mean and variance) can be used to compare cultural/behavioural norms between groups. Importantly, these frequencies can feed back to influence individual decisions, such that a person with similar individual characteristics may make radically different decisions in different communities, depending on what others do.

Formal modelling shows that the average level of education in a community can theoretically alter the cultural dynamics of fertility decline, by modifying the social channels (modes) of communication and changing the rates by which fertility preferences are socially transmitted [33–35]. For example, higher rates of horizontal (peer-to-peer) and oblique (e.g. teacher-to-pupil) transmission may speed up the diffusion of low fertility norms even in populations that are homogeneously high in fertility, less economically developed and/or subject to natural selection pressure [33–35]. Increased frequencies of social interaction with more highly educated people and with non-kin, who are assumed to promote anti-natal values, are predicted to have a fertility-reducing effect [32,36]. Higher average education in a community increases the chances that any person, irrespective of their own characteristics, is exposed to low-fertility behaviour, to contraceptive users or to other peoples’ preferences for educating children [31–36]. The level of education in a community can therefore alter the speed of fertility decline by subtly affecting who we interact with, and thus the preferences we are likely to encounter.

These cultural dynamics also theoretically work at a between-group level (i.e. between neighbouring populations or communities of a larger metapopulation). High rates of social transmission are predicted to homogenize behavioural norms within particular groups [9,37,38], leading to between-group variation in the speed of fertility decline. This can expose individual communities themselves to low-fertility norms while at lower levels of education than would be expected if they were isolated [35]. Cultural evolutionary processes can therefore potentially explain two important patterns that have been observed by demographers. First, fertility declines often differ among neighbouring populations at similar levels of economic development [4,17,18], especially when there are social or cultural boundaries to social transmission, such as linguistic, religious or ethnic affiliations. Second, and conversely, fertility declines can spread across economic boundaries when neighbouring regions share a language or religion [17,18,39]. Examining which mechanisms drive these patterns requires data at the appropriate level of aggregation and a representative sample of communities.

**Figure 1.**
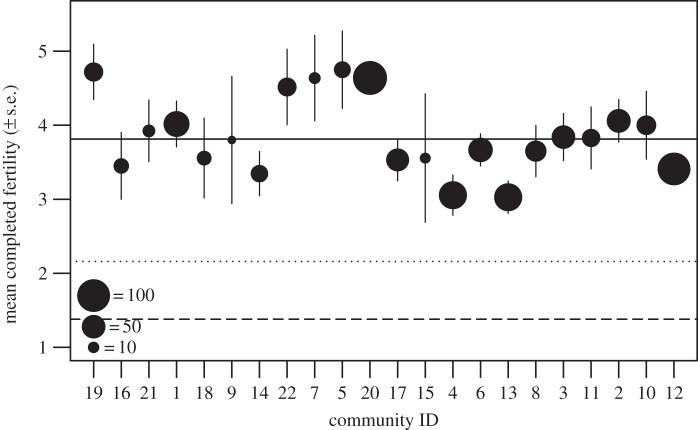
Raw data showing between-community variation in completed fertility ± s.e. (*n* = 907 women aged 45+). Grand mean completed fertility ± s.d. is 3.81 ± 2.15 (solid line). Fertility is high relative to the cohort TFR for the most relevant (1949) birth cohort (2.16, dotted line), and to the national TFR when the data were collected in 2010 (1.38, dashed line). Community ID numbers give the order of sampling, with communities shown in order of increasing population density from left to right. There is no clear trend for decreased fertility in denser communities. The size of each point indicates the sample size of post-reproductive women. Note that group 12 is the town.

In this study, we show that the average level of education in a community substantially accelerates fertility decline independent of individual- and other community-level characteristics, most likely by altering the social interactions of less educated women and thereby the types of fertility norms they encounter. We examined multi-level effects of education on both fertility decline and social network structure and composition, using new data from a randomized study of 1995 women living in 22 randomly selected communities (21 villages and one town) in a rural agricultural area of Poland. Our data provide a rarely observed but valuable insight into a mid-transitional population and our samples are representative of the villages women inhabit (see electronic supplementary material). Under-five mortality is low (see electronic supplementary material, table S1) and completed fertility is high (grand mean = 3.81 (±s.d. 2.15); figure 1), varying significantly across communities from an average of 3.03 (±s.d. 1.85) to 4.75 (±s.d. 2.56) children per woman at the end of her reproductive career (ANOVA, *p* = 0.024; see figure 1 for raw data). Completed fertility is high relative to the Polish total fertility rate (TFR) when the data were collected (1.38 in 2010; electronic supplementary material) and to the TFR for the most relevant birth cohort (2.16 in 1949). Given that Poland is a developed European country, where fertility decline began around 1910 and which has had a below replacement TFR since the early 1990s [40], such high and varied fertility is remarkable.

Our study communities are characterized by centuries of peasant subsistence farming, but are now rapidly transitioning to exclusive dependence on labour-market activities. More than 65% (*n* = 1255) of respondents continue to live in subsistence farming households. The communities are geographically proximate (within an area of approx. 30 km^2^), at effectively equivalent levels of economic modernization, and are highly homogeneous with respect to religion and ethnicity. This enables us to isolate the effects of education from religious or ethnic differences between communities—this is important because fertility declines tend to spread among co-religionists and language speakers independent of economic modernization [17,18,39]. In addition to individual variation, our data capture community-level variation in population size and density, proportion of inhabitants engaging in subsistence farming, average household material wealth and market integration (Material and methods; electronic supplementary material, table S1).

We test a number of predictions designed to disentangle the contribution of cultural transmission hypotheses from purely individualistic optimizing models of fertility decline. If cultural transmission models are correct, then: (i) higher average levels of education in the community, rather than other community-level measures of economic modernization, such as wealth, market integration or population density, should accelerate fertility decline over and above individual characteristics; and (ii) less educated individuals should have lower fertility when living among a higher frequency of highly educated women. Women in highly educated communities should interact with (iii) more highly educated network partners, (iv) more sources of peer-to-peer (horizontal) rather than parent-to-child (vertical) transmission or (v) more non-kin. On the other hand, if individuals make reproductive decisions that are optimal relative to their own characteristics, then: (vi) variation in fertility should decrease along with variation in education; and (vii) community-level education should matter more for highly than poorly educated women, because factors like better educational opportunities, higher employment rates and more intensive reproductive competition may provide ‘runaway’ incentives and advantages to investing in ‘quality’ over quantity [2,11–16].

We standardized our measures to enable comparison at different levels of analysis. Our measures were also group-mean centred so that an individual's score represents her deviation from her community mean (i.e. her education relative to others in her local context rather than to others in the general population—this differs from most previous work, which compares randomly sampled individuals across a wider area). We then use group means to measure community effects—this has the advantage of cleanly partitioning the variation in the effects into within- and between-community components (see electronic supplementary material).

## 2. Results

Education was universally associated with reductions in fertility. Surprisingly, though, individual education was not a significant predictor in all communities, and the effect was larger at the community than at the individual level. A 1 s.d. increase in individual education was associated with a 13% reduction in fertility (95% CI (−0.19, −0.08), Wald *z*-test, *p* < 0.001; electronic supplementary material, table S3), when considering the average (‘fixed’) effect across all communities. This association varied by community and was not always significant, ranging from a 6 to a 20% decrease in fertility for every 1 s.d. increase in education (see electronic supplementary material, table S4).

**Figure 2.**
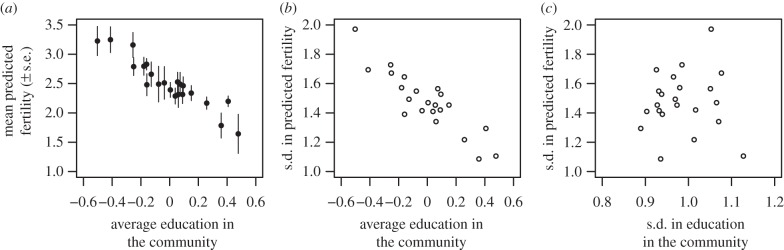
Community-level education is associated with a reduction in (*a*) mean (±s.e.) and (*b*) variance (given as s.d.) in fertility, independent of individual differentials and of controls for between-community variation (*n* = 1972). However, there is no clear relationship between (*c*) variance (given as s.d.) in fertility and variance (given as s.d.) in education. In (*a*) and (*b*), each interval on the *x*-axis corresponds to 1 s.d. in average education. All panels show model-adjusted relationships.

Independent of individual factors, a 1 s.d. increase in average education in the community was associated with an 18% reduction in individual fertility (95% CI (−0.26, −0.10), Wald *z*-test, *p* < 0.001; electronic supplementary material, table S3; figure 2*a*). A 1 s.d. increase in education at the community level therefore had a 1.3 times larger effect than a comparable increase at the individual level. Figure 2*a* shows that, independent of their own characteristics relative to others, women in the most educated community were predicted to have fewer than half as many children (1.64 ± s.e. 0.33) as those in the least educated community (3.25 ± s.e. 0.22). Note that these results are independent of community-level controls for modernization and a range of individual-level controls (see the electronic supplementary material).

Family sizes were more homogeneous in more highly educated communities. Figure 2*b* shows that the variance in predicted fertility (here, given as s.d. to retain the same units) declined as average education in the community increased, and these variances differ significantly across communities (Brown–Forsythe test, *p* < 0.001).

An intuitive explanation of this pattern might be that variance in fertility decreases along with variance in education. However, figure 2*c* shows clearly that this pattern of convergence has no relationship with how varied educational levels were in the community: family sizes were more similar independent of variation in individual education. This convergence is not predicted by purely economic approaches that rely on individual characteristics as the primary drivers of fertility decline.

However, evolutionary-economic approaches do often argue that there may be ‘runaway’ benefits to investing in ‘quality’ over quantity in more educated populations, or reproductive competition to obtain the perceived fitness pay-offs to producing fewer high-quality children [2,11–16]. We examined this possibility by modelling a cross-level interaction between individual and average community education, but this revealed no significant relationship, and the model fit was not improved by including the interaction term (see electronic supplementary material, table S3). This suggests that between-community differences in fertility are not driven by the fertility-reducing behaviour of highly educated women. In other words, highly educated women do not necessarily reduce fertility more dramatically when the local context is conducive to greater investments in offspring education.

**Figure 3.**
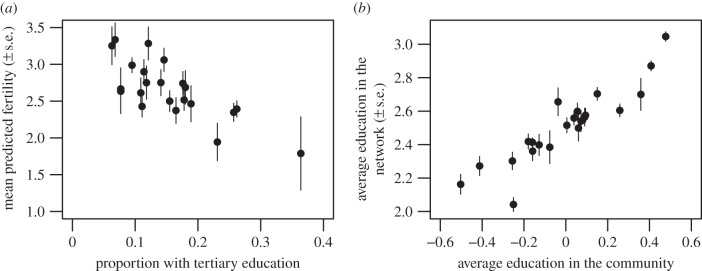
Community education effects on less educated women only. (*a*) Fertility decline is more dramatic among less educated women when the proportion of tertiary-educated women in the community is larger (*n* = 1667), and (*b*) less educated women living in highly educated communities have more educated social network partners (*n* = 1667). Both panels show model-adjusted relationships.

Instead, it appears that social transmission may drive the differences between our study communities. Dividing each community into high and lower education brackets, with ‘high’ representing individuals with tertiary education and ‘lower’ representing everybody else, we re-examined the predicted fertility scores of only the lower-educated women, against the proportion of tertiary-educated women in the 22 communities. This allows us to examine how lower-educated women, who are not expected to reduce fertility substantially based on their individual characteristics, may be altering their reproductive strategies based on what other women around them do. As figure 3*a* shows, less educated women had lower fertility in communities with a higher proportion of tertiary-educated women. This implies that simply living among a higher frequency of tertiary-educated women may have influenced less educated women to reduce their own fertility.

What are the potential mechanisms driving these effects? Cultural evolutionary theory suggests that the level of education in a community may independently drive changes in ego-network size, density, proportion of alters who are kin, proportion of alters who are horizontal sources of transmission (neither parents nor individuals from the parental generation) and average education of alters. We tested these hypotheses using the same individual- and community-level controls, but found no evidence that community education is associated with social network changes in size, density, proportion of alters who are kin or the proportion who are horizontal sources of transmission (see electronic supplementary material, table S6). However, network alters were significantly more educated when ego lived in a highly educated community. Independent of ego's education, every 1 s.d. increase in community-level education was associated with a 12% increase in average education among the alters in the network (95% CI (0.04, 0.21), *t*-test, *p* = 0.006; electronic supplementary material, table S6). Figure 3*b* shows that this effect holds true when focusing again on less educated women only; alters in the network were significantly more educated when ego lived in a community with higher average education. This clearly indicates that simply living in a highly educated community may influence the characteristics of the friends that women choose to interact with, and thus the kinds of social information they are likely to receive. It is important to note that 48% of all network partners were not resident in the same community as ego (see electronic supplementary material, table S2). This result thus shows that less educated women have strong ties with more highly educated women both within and beyond the boundaries of their local communities.

## 3. Discussion

Individual characteristics are only part of the story of demographic transition, and studies that exclusively rely on them are likely to underestimate the importance of cultural transmission in driving fertility decline. Cultural norms are by definition factors beyond the individual; explicitly measuring and analysing community-level characteristics is therefore crucial for testing hypotheses about the local cultural transmission of fertility preferences. Community-level characteristics, for example the frequency of highly educated women, do not simply tell us about the distribution of resources and opportunities in a population; they themselves may drive interesting and important social dynamics, which cannot be captured using individual characteristics alone. While demographers have long been interested in measuring contextual effects on reproductive behaviour, these have not often been measured with appropriate community-level data and thus could not determine whether community effects existed at socially relevant levels of aggregation, or indeed if they were independent of alternative predictors of local community modernization. Our study shows that they are, and furthermore examines some of the causal pathways by which average education might feed back to influence individual behaviour.

Among 22 groups of a culturally and ethnically homogeneous population at effectively equivalent levels of economic modernization, the education level of other people in the community affected individuals’ reproductive outcomes. Indeed, the community average had a larger effect than the individual effect; a poorly educated woman was predicted to have twice as many children when living in a less educated community compared with living in a highly educated one.

Family sizes were also less varied in highly educated communities, but this lack of variation in fertility was not due to homogeneity in educational levels. Reproductive norms can therefore converge on a small family size independent of individual variation in education. Thus, although highly educated women do tend to have lower fertility than less educated women within communities, between-community differences were driven by the behaviour of less-educated women only. It follows that comparing individual characteristics across large swathes of a population will be insufficient for a full understanding of the mechanisms of fertility decline.

Less educated women had strong ties with significantly more educated women, both within and beyond their communities, when surrounded by more educated neighbours—this result is all the more striking because the other social network characteristics do not appear to change with community education. Any person in a more educated group is exposed to a greater variety of social learning models, as well as a greater number of individuals exhibiting low-fertility behaviour. Increasing average education may therefore alter both the content and structure of social interactions, consistent with cultural evolutionary models assuming that it leads to higher rates of social transmission, and thereby speeds up the diffusion of low fertility norms [31–36]. Interestingly, the fact that networks contained highly educated alters living outside the immediate community also points to a mechanism for spreading low-fertility norms between communities: higher average education within a community leads to interaction with more educated women across communities—this is consistent with formal modelling [35].

Our results strongly suggest that social transmission from highly to poorly educated women accelerates the pace of fertility decline, once a critical mass of educated women is reached. The principal mechanism driving this effect does not appear to be a greater presence of non-kin [36] or of horizontal sources of transmission [33–35] among the strong ties of ego-networks, but rather an increased frequency of interaction with more highly educated women. The individual adoption of low fertility norms and behaviour may therefore be prestige- or conformist-biased [32,37,38,41], or indeed simply the result of a more neutral process of randomly copying the most frequent behaviour in the population [31,32]. Less educated women in more educated communities are more likely to interact with and observe the reproductive behaviour of highly educated neighbours and friends, who may be more likely to endorse behaviours consistent with lower fertility. This exposure may change the expectations that less educated women have about the most appropriate behaviour in their local community, while also legitimizing and facilitating them to change their reproductive preferences within their private social networks. This can then contribute to a faster fertility decline in the community and lead to between-community variation in the pace of demographic transition. It is important to note both that the predicted differences in fertility between these geographically proximate communities are large, and that the ego-networks reported here are mainly distributed in the local villages and towns (see electronic supplementary material, table S2). District- or province-level analysis that aggregates across these kinds of small-scale social structures cannot adequately capture these dynamics.

We do not wish to imply that rational-actor decision-making is unimportant in driving fertility decline, or that cultural transmission mechanisms are the only important causes of this phenomenon. On the contrary, our results demonstrate the need to integrate theory and evidence on cultural dynamics at the group level with individual decision-making that optimizes the perceived costs and benefits to reducing fertility. We simply emphasize that those perceptions are partly driven by cultural dynamics beyond the individual, with important implications for the speed of fertility decline. The approach outlined here provides a mechanistic link between the individualist, cost–benefit decisions that people appear to make, and which a plethora of micro-level data support, and the larger ideational shifts that make smaller family sizes acceptable and desirable. Irrespective of whether explicit ‘norms’ are being transmitted or whether individuals simply respond to the easily observable correlation between fewer children and higher social status, social transmission of fertility decline should homogenize behaviour within groups [9,37,38]; which is a prediction that our data supports. Whether the initial fertility-reducers in a population are responding to the real or perceived economic costs of children is essentially a different question to whether cultural transmission can spread low-fertility norms, and our analysis focuses only on the latter question. All that is needed for the process to take off is that a certain proportion of individuals invest in education and that education is reliably associated with lower fertility. Individuals do not need to know what the mean level of education is in their community for this characteristic to affect their probability of encountering low-fertility norms and behaviour. Community-level characteristics can alter the social cues to the most appropriate behaviour in ways that are neither predicted by a person's individual characteristics nor by the level of economic or structural modernization in a particular group. Community-level factors may therefore have independent causal force [35].

These results raise important theoretical questions about the extent to which individuals are influenced by their neighbours in perhaps the most important decisions of their lives—how many children to have—decisions that are expected to be under selection pressure [42]. Our findings also have important implications in mid-transitional populations. Apart from the fact that improving average education in a community will accelerate fertility decline, even among less educated individuals [19–26], our results imply that the mean may be more important than the mode. It is possible that universally high education may not be as important as ensuring that a critical mass of highly educated women is reached, by which point the changing structure of social transmission will facilitate the diffusion of low-fertility norms to all individuals in the community. That feedback between community- and individual-level education ratchets up the speed of fertility decline may also have significant implications for global fertility projections. More fine-grained data and multi-level analysis are needed to explicitly test hypotheses about the mechanisms driving these effects. Although only one study to date has examined the influence of education on fertility at multiple higher levels of aggregation [20], more local contexts mattered most and our study supports this assertion. Thus, in the era of ‘big data’, there remains an important place for smaller-scale anthropological demographic research in elucidating the mechanisms that underlie fertility change.

## 4. Material and methods

Data were collected between 2009 and 2010 using a standardized anonymous questionnaire and structured interview (see the electronic supplementary material for further discussion). We used Poisson, linear and binomial multi-level regression [43] techniques, which explicitly account for the non-independence of individuals living in the same local context, to simultaneously model the effects of individual- and community-level education on (i) fertility, and on the following social network characteristics of (ii) size, (iii) density, (iv) proportion of kin, (v) proportion of horizontal sources of transmission and (vi) average education of network partners, excluding ego (see the electronic supplementary material, tables S3–S6). At the individual level, all models included controls for age, farmer status, household material wealth and household market integration. In the fertility analysis, we additionally controlled for age squared and experience of under-five mortality, as well as including an additional random term at the individual level to account for over-dispersion in the data. At the community level, all models included controls for population density, proportion of the population engaging in farming, average household material wealth and average household market integration (see the electronic supplementary material). These are important controls for alternative measures of modernization at the relevant local levels of analysis because all of these factors might jointly drive higher education and lower fertility preferences.

We examined the following alternative model specifications to check the robustness of our results in predicting fertility outcomes. None of these altered our findings (see electronic supplementary material, table S5): (i) excluding unmarried women (*n* = 374) from the analysis, (ii) including nonlinear parametrizations of individual education on fertility within communities, (iii) using uncentred variables, (iv) removing non-significant variables or substituting average education for any other community-level predictor; the model was a significantly worse fit whenever we excluded average community education.

## Notes

Informed consent was obtained from all participants and the study protocols were approved by the Ethics Committee of the Department of Anthropology at University College London.
